# Factors associated with the duration of hospitalisation among COVID-19 patients in Vietnam: A survival analysis

**DOI:** 10.1017/S0950268820001259

**Published:** 2020-06-10

**Authors:** Pham Quang Thai, Do Thi Thanh Toan, Dinh Thai Son, Hoang Thi Hai Van, Luu Ngoc Minh, Le Xuan Hung, Ngo Van Toan, Luu Ngoc Hoat, Duong Huy Luong, Luong Ngoc Khue, Nguyen Trong Khoa, Le Thi Huong

**Affiliations:** 1 The National Institute of Hygiene and Epidemiology, Hanoi, Vietnam; 2 Department of Biostatistics and Health Informatics, Institute of Preventive Medicine and Public Health, Hanoi Medical University, Hanoi, Vietnam; 3 Department of Environmental Health, Institute of Preventive Medicine and Public Health, Hanoi Medical University, Hanoi, Vietnam; 4 Ministry of Health, Hanoi, Vietnam; 5 Institute of Preventive Medicine and Public Health, Hanoi Medical University, Hanoi, Vietnam

**Keywords:** COVID-19, duration of stay, survival, Vietnam

## Abstract

**Background:**

The median duration of hospital stays due to COVID-19 has been reported in several studies on China as 10−13 days. Global studies have indicated that the length of hospitalisation depends on different factors, such as the time elapsed from exposure to symptom onset, and from symptom onset to hospital admission, as well as specificities of the country under study. The goal of this paper is to identify factors associated with the median duration of hospital stays of COVID-19 patients during the second COVID-19 wave that hit Vietnam from 5 March to 8 April 2020.

**Method:**

We used retrospective data on 133 hospitalised patients with COVID-19 recorded over at least two weeks during the study period. The Cox proportional-hazards regression model was applied to determine the potential risk factors associated with length of hospital stay.

**Results:**

There were 65 (48.9%) females, 98 (73.7%) patients 48 years old or younger, 15 (11.3%) persons with comorbidities, 21 (16.0%) severely ill patients and 5 (3.8%) individuals with life-threatening conditions. Eighty-two (61.7%) patients were discharged after testing negative for the SARS-CoV-2 virus, 51 were still in the hospital at the end of the study period and none died. The median duration of stay in a hospital was 21 (IQR: 16–34) days. The multivariable Cox regression model showed that age, residence and sources of contamination were significantly associated with longer duration of hospitalisation.

**Conclusion:**

A close look at how long COVID-19 patients stayed in the hospital could provide an overview of their treatment process in Vietnam, and support the country's National Steering Committee on COVID-19 Prevention and Control in the efficient allocation of resources over the next stages of the COVID-19 prevention period.

## Introduction

In December 2019, Chinese public health authorities reported several cases of the acute respiratory syndrome in Wuhan City, Hubei province, China [1]. The disease is now referred to as the coronavirus disease 2019 (COVID-19), and the causative virus is called severe acute respiratory syndrome coronavirus 2 (SARS-CoV-2) [1]. In January 2020, the first cases of COVID-19 were identified in Vietnam [2] among people who had returned from Wuhan, the centre of the COVID-19 pandemic in China. In early April 2020, the Vietnamese government declared COVID-2019 a nationwide epidemic [2] with more than 200 confirmed cases of COVID-19 and no deaths recorded.

From January to February 2020, COVID-19 spread throughout Vietnam with different degrees of severity, ranging from mild to critical. In a large cohort of more than 44 000 persons with COVID-19 in China, 81% were diagnosed with a mild-to-moderate illness (i.e. mild symptoms up to mild pneumonia); 14% were severe cases (i.e. dyspnoea, hypoxia or >50% lung involvement on imaging); and 5% were in critical condition (e.g. respiratory failure, shock or multiorgan system dysfunction) [3]. In another study from the United States on confirmed cases of COVID-19, the proportion of hospitalised patients was 19%, and the proportion of persons admitted to intensive care units (ICU) was 6% [4].

Given the continuing spread of the SARS-CoV-2, healthcare systems and healthcare workers in many countries including Vietnam are facing a multitude of challenges at all stages of the global COVID-19 pandemic [5]. A thorough preparation for the outbreak of COVID-19 is extremely important, especially for high-risk countries. Learning about the duration of hospitalisation among COVID-19 patients and its associated factors could provide a better understanding of its impact on medical interventions as well as hospital capacities to cope with the surge of COVID-19 patients. To date, studies of COVID-19 mostly focused on epidemiological investigation, prevention and control, diagnosis and treatment [3, 6–8]. Fewer studies have investigated the duration of COVID-19 patients' hospital stays during the epidemic. The median duration of stay due to COVID-19 has been reported in several studies of China as 10−13 days [9–11]. However, the length of stay depends on various factors, such as the time elapsed from exposure to symptom onset, and from this onset to the time of hospital admission, as well as various factors related to the country-specific context. The primary goal of this study is to estimate the duration of hospital stay and identify its associated factors among patients admitted with COVID-19 in Vietnam.

## Method

### Study design and setting

As of 8 April 2020, Vietnam reported 251 cases of COVID-19 infection. The COVID-19 pandemic in Vietnam was divided into two phases. Phase one had 16 cases related to the Wuhan outbreak that had been completely treated. After 21 days without any new reporting of cases, phase two began with the identification, on 5 March, of the 17th COVID-19 patient who returned from Europe a few days before. In this study, we examine the median duration of hospital stay during this second phase of the COVID-19 pandemic, from 5 March to 8 April 2020.

### Study outcome

On the basis of hospital discharge data published by the Vietnam Ministry of Health by 8 April 2020, the median duration of hospital stays among 251 COVID-19 patients was 16 days. The study participants were enrolled over a period, and the study endpoint was a specific date. Thus, patients who enrolled later were followed for a shorter time than patients who enrolled early. To reduce errors in estimating the duration of hospital stay, we excluded patients whose hospitalisation duration was recorded for 2 weeks before 8 April 2020. This resulted in a final sample of 133 confirmed COVID-19 patients. Data on epidemiological and demographic characteristics, and hospital discharge rates, were extracted from published data of the National Steering Committee on COVID-19 Prevention and Control and the Ministry of Health.

A case of COVID-19 was defined as having a positive result on real-time reverse transcriptase-polymerase chain reaction (RT-PCR) assay of nasopharyngeal swab samples. The duration of hospital stay was calculated as the discharge date minus admission date. Patients with COVID-19 were discharged from the hospital if they had two consecutive negative RT-PCR tests on different days and no longer showed any symptoms.

### Independent variables

We obtained the following demographic characteristics: age (i.e. less than or equal to 48 years of age, or older than 48 years old; the cutoff was based on the third quartile of the age variable in all 251 cases recorded in Vietnam); gender (i.e. male or female); occupation (i.e. working in a hospital or not) and region of residence (i.e. North, Central or South). Epidemiological information included pre-treatment quarantine status (i.e. with or without quarantine). Binary variables were used including clinical symptoms of COVID-19, coexisting conditions and severe conditions. We also obtained a variable on sources of contamination classified into three groups: i.e. those who had returned from abroad, cases with pinpointed sources of infection or cases identified in the country.

### Statistical analysis

Demographic and epidemiological variables were described by frequency and percentage. Comparing the difference between demographic and epidemiological variables and the outcome variable was performed by *χ*^2^ tests. The Kaplan−Meier method and log-rank test were used to estimate the cumulative probability of hospital discharge by each independent variable. The log-rank test compared the survival distributions of hospital duration for all epidemiological and demographic characteristic variables. The Cox proportional-hazards regression model was applied to determine the potential risk factors associated with hospital discharge. Variables for the Cox regression model were selected using the leaps-and-bounds algorithm and based on Akaike information criterion (AIC) and Bayesian information criterion (BIC). Statistical significance was defined as *P* < 0.05. Statistical analysis was conducted using Stata and R software version 3.6.3.

The Vietnamese Ministry of Health agreed to this retrospective study.

## Results

Table 1 describes the epidemiological and demographic characteristics of the 133 patients in our sample. Among 133 cases with confirmed SARS-CoV-2 infection, 82 patients had been discharged by the end of the study. Patients less than or equal to 48 years of age accounted for 73.7% of the patients. There were 65 (48.9%) females and four patients who were working in hospitals. The percentage of patients who lived in the North, Central and South regions of Vietnam was 49.6%, 15.8% and 34.6%, respectively. A history of exposure to SARS-CoV-2 from abroad was found in most patients (72.9%). Half of the patients were identified as being infected by COVID-19 while in isolation facilities in Vietnam (55.6%). The proportion of patients who had common symptoms at the onset of illness, such as fever, cough and fatigue, accounted for 66.9%. Only 11.3% of the patients had comorbidities. Eighty-two patients were discharged (by the endpoint of the study period). There was a statistically significant difference in the occupation, area of residence and sources of infection between patients who were discharged and those who were not.

**Table 1.** Epidemiological and clinical characteristics

	Presence of endpoint		
	No	Yes	All
	(*n* = 51)	(*n* = 82)	(*n* = 133)
Age			
Median (Q1, Q3)	29.0 (22.0, 54.0)	29.0 (22.0, 43.0)	29.0 (22.0, 50.0)
Time			
Median (Q1, Q3)	19.0 (17.0, 22.0)	16.0 (14.0, 19.0)	17.0 (16.0, 21.0)
Gender			
Female	25 (49.0%)	40 (48.8%)	65 (48.9%)
Male	26 (51.0%)	42 (51.2%)	68 (51.1%)
Age group			
⩽48	34 (66.7%)	64 (78.0%)	98 (73.7%)
>48	17 (33.3%)	18 (22.0%)	35 (26.3%)
Occupation			
Other careers	47 (92.2%)	82 (100.0%)	129 (97.0%)
Working at hospitals	4 (7.8%)	0 (0.0%)	4 (3.0%)
Living area			
North area	36 (70.6%)	30 (36.6%)	66 (49.6%)
Central area	2 (3.9%)	19 (23.2%)	21 (15.8%)
South area	13 (25.5%)	33 (40.2%)	46 (34.6%)
Outbreak			
Foreign outbreak	31 (60.8%)	66 (80.5%)	97 (72.9%)
Identified cases	8 (15.7%)	14 (17.1%)	22 (16.5%)
Domestic outbreak	12 (23.5%)	2 (2.4%)	14 (10.5%)
Quarantine			
Yes	29 (56.9%)	45 (54.9%)	74 (55.6%)
No	22 (43.1%)	37 (45.1%)	59 (44.4%)
Symptoms			
No	20 (39.2%)	24 (29.3%)	44 (33.1%)
Yes	31 (60.8%)	58 (70.7%)	89 (66.9%)
Coexisting diseases			
No	46 (90.2%)	72 (87.8%)	118 (88.7%)
Yes	5 (9.8%)	10 (12.2%)	15 (11.3%)
Severity of COVID-19			
No	36 (73.5%)	74 (90.2%)	110 (84.0%)
Yes	13 (26.5%)	8 (9.8%)	21 (16.0%)

**Fig. 1.**
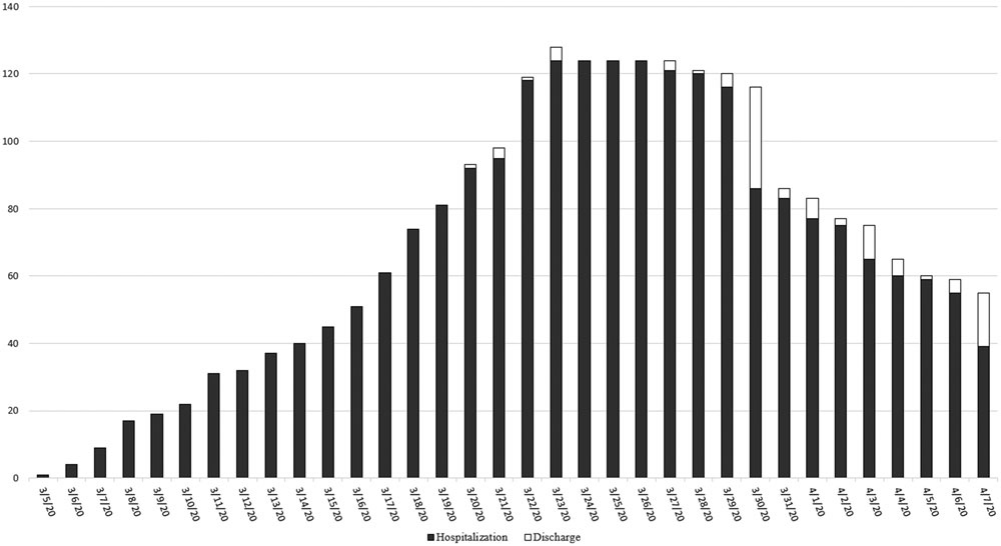
Number of patients admitted and discharged.

Figure 1 shows that the number of hospitalised patients increased gradually from 5 March 2020, and peaked at 124 patients on 23 March 2020. The first case was discharged from the hospital after 15 days. The maximum number of patients discharged in a day was 30 people.

**Fig. 2.**
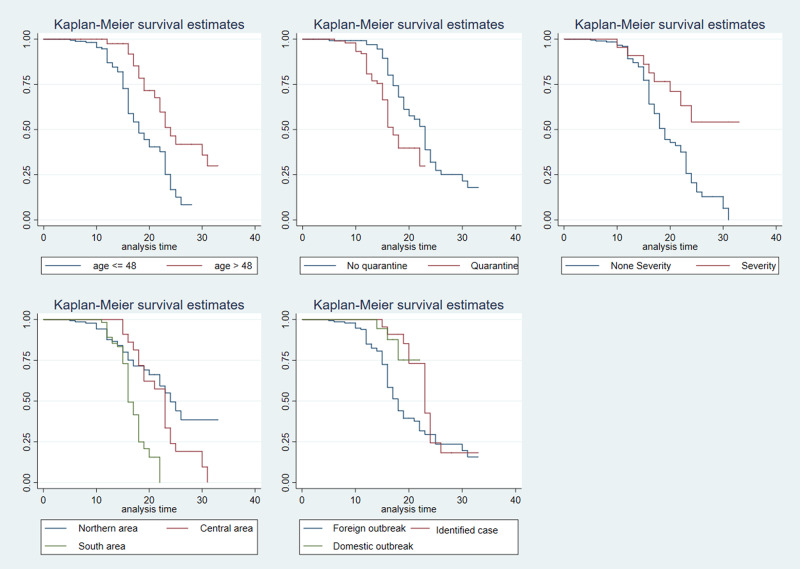
Risk factors for time to hospital discharge by univariable Kaplan−Meier survival analysis.

The log-rank test results indicated statistically significant differences in the duration of hospital stay by age group, source of infection, quarantine before treatment, severe cases and region of residence (Fig. 2). The median duration of hospital stay for the group less than or equal to 48 years of age was 18 days, while it was 24 days for the group over 48 years old. The probability of discharge over time among cases that had been infected within Vietnam itself (and by someone identified as having COVID-19 or not) was higher than for the patients infected abroad. This probability in the isolated group was lower than that of the non-isolated group. The median hospitalisation duration of patients from the North and Central regions of Vietnam was higher than that for the South.

**Fig. 3.**
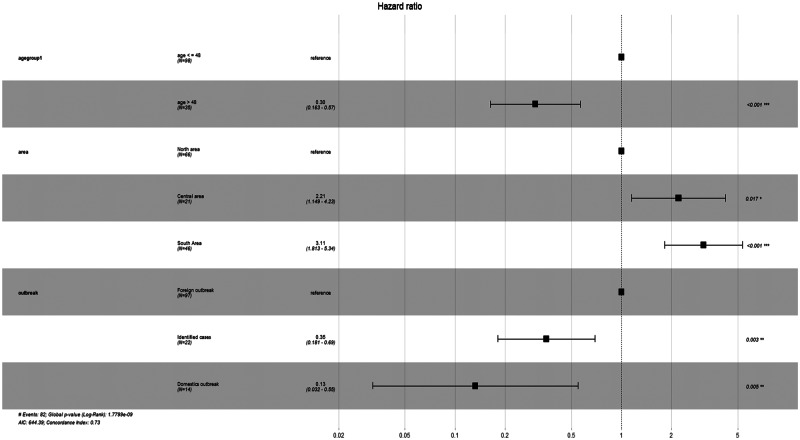
Risk factors for longer hospitalisation of COVID-19: hazard ratios from Cox regression model.

Figure 3 describes the results of the Cox regression model to identify the factors associated with longer hospitalisation for COVID-19. The model with the lowest AIC and BIC contained three variables, including age group, region of residence and source of infection. The hazard ratio of age groups indicated that compared to patients aged less than or equal to 48 years, those older than 48 years old had a 70% increase in the risk/hazard of continued hospital treatment. Compared to the foreign infected group, patients who had been infected inside Vietnam had an increased risk/hazard of longer hospitalisation of 65% and 87%, respectively. Patients living in North Vietnam had a higher hazard of longer hospitalisation duration.

## Discussion

Among 133 COVID-19 patients hospitalised during the period from 5 March to 8 April 2020, in Vietnam, 82 patients (61.7%) were discharged home alive and 51 were still in the hospital at the end of the study period. The age of patients in our study ranged from 2 to 88 years and nearly half of the patients (48.9%) were females. Similar to a study in Beijing and some other areas in China [3], there was a wide age range, i.e. from 1 to 94 years of age, and a slight gender difference among hospitalised patients with COVID-19. It is suggested that people of all ages and sexes could be susceptible to COVID-19. Also, similar to another study in Singapore [12], half of the patients in our study were linked to tour groups, a conference or students studying abroad, and half were identified in quarantine areas.

The median duration of hospitalisation among our patients was 21 (IQR: 16–34) days. A recent study in Sichuan province, China, also showed a similar duration of stay for all confirmed inpatient cases of 19 (IQR: 3–41) days. Nevertheless, in the United States and several European countries, the duration of hospital stay is shorter with an average of 7–8 days [4, 13, 14]. This difference could be explained by a difference in strategies for the prevention and control of COVID-19.

As of mid-April 2020, when the number of COVID-19 infections reached nearly 2 million patients globally, Vietnam was still successful in its disease prevention and control efforts, with only 260 confirmed cases of COVID-19 [2, 15]. Given lessons learned from successfully battling the SARS epidemic 2003, Vietnam implemented two decisive measures in containing the spread of COVID-19, including aggressive contact tracing and strict monitoring of the quarantine of suspected infections [16]. If a person enters Vietnam from virus-hit countries, or a person comes in close contact with a confirmed case, she/he will be quarantined in publicly managed facilities and will be tested with real time RT-PCR for coronavirus. So far, 300 000 tests have been conducted. All patients with positive results will be referred to medical facilities and discharged from the hospital only when there is no sign of fever for three days and tests prove negative twice in three days. In this study, about half of the admitted patients had no symptom onset because of this early detection strategy, and discharged patients had been tested as negative three consecutive times. This could be a possible explanation for the relatively long length of hospital stay among the patients in our sample.

Similarly, although Sichuan province is located near Hubei province, where the transmission of COVID-19 originated, the province had reported less than 600 cases only as of March 2020 due to a series of steps towards epidemic prevention and control measures taken by Sichuan government authorities and residents' quarantine measures [17]. In contrast, in the United States or several European countries where the average duration of stay for COVID-19 patients was shorter, suspected or confirmed patients with mild symptoms were being encouraged to self-isolate and guided in self-care at home through consultation networks or telemedicine systems. Therefore, people admitted to hospitals were often in severe or critical condition. In a study in the UK among 16 479 patients admitted to hospital with COVID-19, the median duration of symptoms before admission was four days with the most common recorded comorbidities being chronic cardiac disease (29%), diabetes (19%), chronic pulmonary disease (19%) and asthma (14%) [14].

In this study, we identified several influential factors in the duration of hospital stay of discharged COVID-19 patients. After variable selection based on AIC and BIC, the results from the Cox regression model showed that patients of more than 48 years, those living in the North, and those whose infection was identified domestically had longer lengths of stay. Older people are more likely to have comorbid conditions and be severe cases, and therefore may require more medical care than the younger. Older age has been found to be the main factor associated with hospitalisation in several studies [3, 4, 13, 14]. In the United States, for instance, people older than 65 had the highest rate of hospitalisation per capita [9]. In our study, 11.3% of patients had coexisting disorders, 16.0% were categorised as severe patients, and 3.8% were in life-threatening conditions, and almost all of them were over 60 years old.

The two largest airports of Vietnam are in Hanoi (in the North) and Ho Chi Minh City (in the South). The government foresaw that an overload of the quarantine facilities in these two cities would soon become an influential factor in efforts to prevent the spread of COVID-19. By mid-March 2020, a decision to suspend all foreign flights to these two airports was officially issued [16]. Therefore, in COVID-19 phase two, most of the hospitalised patients were local and secondary cases. Our study found that local and secondary cases had higher risk/hazard of longer hospital stay compared to COVID-19 patients who returned to Vietnam from abroad. Another possible reason is that people who returned from abroad often had a longer incubation time before entering Vietnam.

As revealed by a study of China, a longer duration of disease/hospitalisation means a greater medical burden, especially when the transmission of COVID-19 might rapidly increase patient volumes, to the point of excessively exceeding healthcare capacities [10]. This applies to the situation when the existing number of patients exceeds the control capabilities. With a population of 95 million, Vietnam is currently ranked 103rd in the world in terms of the number of confirmed COVID-19 cases with zero deaths. This is probably due to preventive measures taken by the government authorities, that have been highly appreciated by the World Health Organization, and that are guided by four principles: (i) timely prevention, isolation and treatment on the spot; (ii) required facilities, equipment, medicine and protective equipment on the spot; (iii) necessary funding on the spot and (iv) supportive human resources on the spot. The model also empowers the lower-level health system and helps them show determination and responsibility for the common good. Given that most patients in Vietnam have been detected at an early stage with mild illness or as asymptomatic, patients could stay longer to receive care in select hospitals to avoid cross-infection and the crowding of medical resources.

### Strengths and limitations

The strength of this study is the use of survival analysis. In survival analysis, censored data are not the same as missing data. Participants who had not completed treatment at the endpoint of the study were not excluded and contributed time at risk to the analysis up to the last interval during which they were in hospitals.

This study was limited by a lack of data on the exposure history of people returning from abroad. If their exposure time was long, it could have affected the length of their hospitalisation in Vietnam.

## Conclusion

The relatively long duration of hospital stays observed among admitted COVID-19 patients in Vietnam could help inform the strategy of the Vietnam government in implementing contact tracing policy in the early detection of suspected cases and thus help prevent larger spread to the community. Age and sources of contamination are the risk factors for longer hospitalisation due to COVID-19.
